# Supplementary material for: Denitrification Characteristics of the Low-Temperature Tolerant Denitrification Strain Achromobacter spiritinus HS2 and Its Application
Source: Microorganisms. 2024 Feb 23;12(3):451. doi: 10.3390/microorganisms12030451 (PMC10971896; doi:10.3390/microorganisms12030451)
Supplement: Supplementary file 1 [file microorganisms-12-00451-s001.zip › microorganisms-2867250-supplementary.pdf]

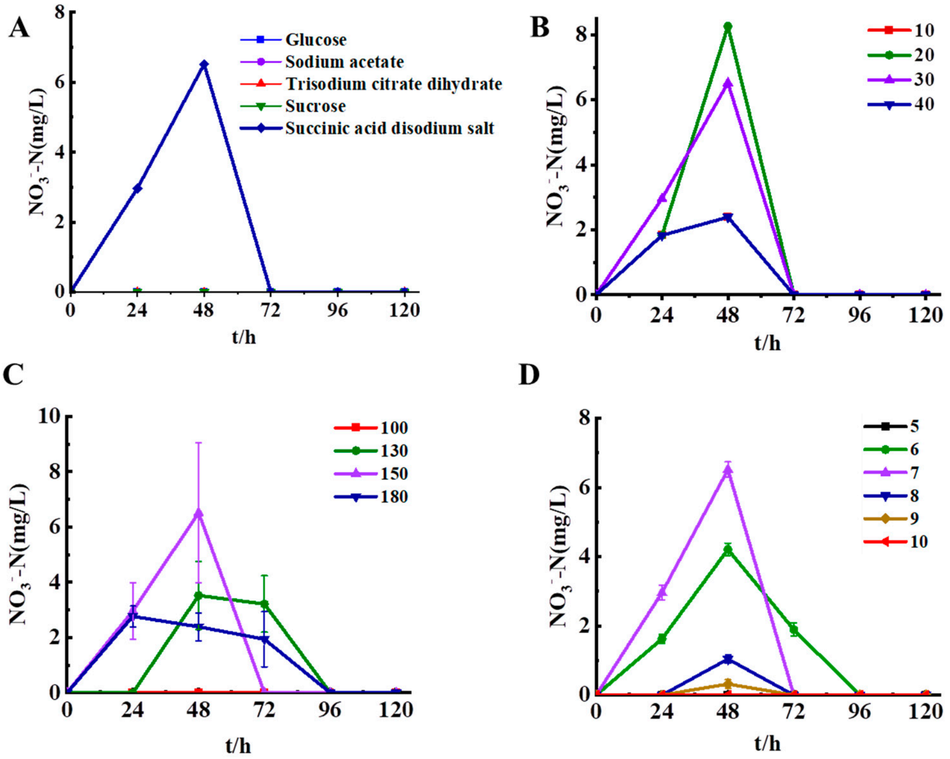

Figure S1 nitrate accumulation of strain HS2 under various factors (A: Carbon source, B: Carbon-to-nitrogen ratio, C: Dissolved oxygen, D: pH)

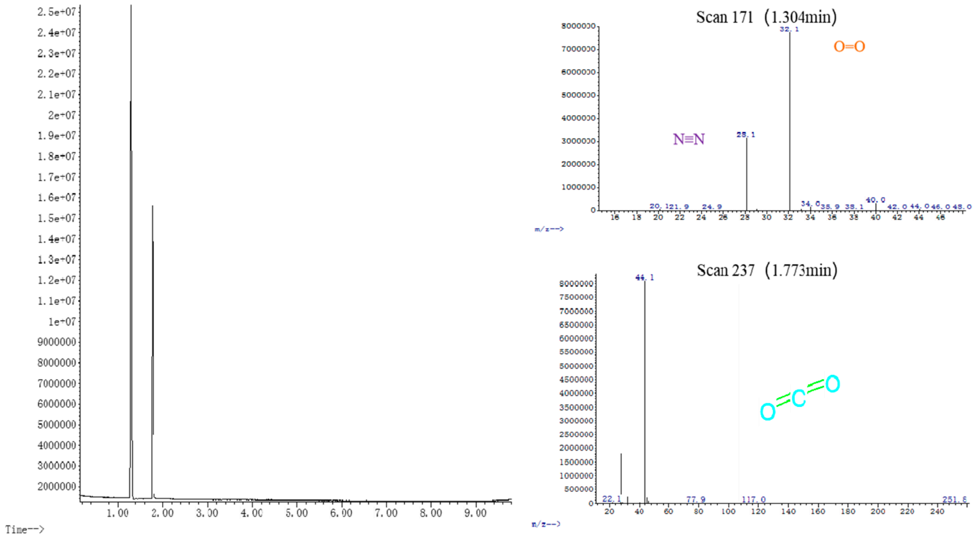

Figure S2 Gas products of strain HS2 in HN-AD
